# Transcriptome Analysis of the Pathogenic Mechanism of the Novel Pathogenic Fungus *Bipolaris fujianensis* in Chinese Fir (*Cunninghamia lanceolata*) Shoot Blight

**DOI:** 10.3390/biology14111488

**Published:** 2025-10-24

**Authors:** Bin Lin, Peiwen Yang, Ruifeng Luo, Ying Lu, Zhe Li, Menglan Shang, Wangdong Xu, Zihui Huang, Guanghong Liang, Qinghua Zhang

**Affiliations:** College of Forestry, Fujian Agriculture and Forestry University, Fuzhou 350002, China; linbin0412@163.com (B.L.); y1119553830@163.com (P.Y.); 52304022046@fafu.edu.cn (R.L.); 18162312296@163.com (Y.L.); 13028752138@163.com (Z.L.); m18537582636@163.com (M.S.); liaoyound709@163.com (W.X.); a1807605023@163.com (Z.H.); fjlgh@126.com (G.L.)

**Keywords:** *Bipolaris fujianensis*, RNA-seq, Chinese fir shoot blight, pathogenicity

## Abstract

**Simple Summary:**

The molecular pathogenesis of *Bipolaris fujianensis*, a novel fungal pathogen causing shoot blight in Chinese fir (*Cunninghamia lanceolata*), remains unclear. Through comparative transcriptomics, we analyzed its gene expression during infection. Key findings include the identification of differentially expressed genes involved in transport, hydrolysis, carbon metabolism, and biosynthesis of secondary metabolites. Notably, we predicted the involvement of cytochrome P450 and a major facilitator superfamily (MFS) transporter in the production and transport of the diterpenoid toxin ophiobolin F. These insights advance the understanding of *B. fujianensis* virulence and may facilitate the development of control strategies against this disease.

**Abstract:**

*Bipolaris fujianensis* is a novel pathogenic species causing Chinese fir (*Cunninghamia lanceolata*) shoot blight (CFSB), first discovered in Nanping City, Fujian Province. However, its molecular pathogenic mechanisms remain largely unknown. Elucidating theses mechanisms has the potential of aiding future developments in disease management and resistance breeding in Chinese fir. In this regard, we examined the expression pattern of *B. fujianensis* grown on PDA (BGPDA), and during infection of Chinese fir at 24 h (BGCF-E), 48 h (BGCF-M), and 5 d (BGCF-L) post inoculation. Comparative transcriptome analysis identified 4133 differentially expressed genes (DEGs), including 1778 upregulated and 2355 downregulated in BGCF compared with BGPDA. During the infection process, Gene ontology (GO) enrichment analysis indicated that transporters and hydrolases in the molecular function categories play essential roles. Kyoto encyclopedia of genes and genomes (KEGG) analyses showed glycolysis/gluconeogenesis, carbon metabolism, and secondary metabolite biosynthesis were the major enriched pathways. Furthermore, this pathogen could produce diterpenoid toxin ophiobolin F, with cytochrome P450 and MFS transport proteins likely involved in its biosynthesis and transport predicted by RT-qPCR.

## 1. Introduction

Chinese fir (*Cunninghamia lanceolata*) is a key afforestation species widely cultivated in Southern China. It is characterized by rapid growth, high-quality wood, excellent productivity, and broad applicability. Moreover, its strong carbon sequestration capacity further enhances its ecological and economic value. However, the expansion of large-scale monoculture plantations has been accompanied by increased disease incidence [1,2]. In recent years, outbreaks of anthracnose, root rot and shoot blight have posed serious threats to the sustainable development of the Chinese fir industry [3,4,5]. Among these, shoot blight is particularly devastating, primarily infecting young shoots and needles, leading to branches and foliage dieback, and ultimately impairing tree growth [6].

Many species of *Bipolaris* are widely distributed fungal pathogens that infect gramineous plants. They cause a variety of typical symptoms, such as leaf spot, leaf blight, and seedling blight, resulting in significant crop yield losses [7,8,9]. Several species, including *B. oryzae*, *B. sorghicola*, *B. maydis*, and *B. zizaniae,* are known to produce non-host-specific toxins, notably ophiobolin (e.g., cochliobolin), which attack host cells and induce cell death [10]. These toxins have been shown to inhibit coleoptile and root growth, seed germination, and seedling development in cereals such as rice, sorghum, and wheat [11,12]. Moreover, ophiobolin A and 3-endosporin A induce brown necrotic spots on the leaves of various plant species [13], and exhibit strong pathogenicity toward wild oats, barnyard grass, maize, and other crops [14]. At low concentrations, ophiobolin A also inhibits the growth of several fungi, including *Gloeosporium laeticolor*, *G. kaki*, *Macrosporium bataticola*, *Glomerella lagenarium*, *Trichophyton purpureum*, and *T. interdigitale* [15].

The newly identified *B. fujianensis* is the causal agent of Chinese fir shoot blight (CFSB), and shows significantly higher pathogenicity on Cupressaceae host than on Gramineae [16]. However, its molecular basis of pathogenicity remains unclear, warranting further investigation. The infection process of microbial pathogens is complex, and often involves the coordinated action of multiple genes. In recent years, high-throughput RNA sequencing (RNA-Seq) has rapidly advanced and become a powerful tool for analyzing pathogen–host interactions and identifying pathogenicity-related genes [17]. Transcriptomic has been successfully applied to investigate pathogenic genes in various pathogenic fungi, including *Colletotrichum truncatum*, *Mycosphaerella fijiensis*, *C. gloeosporioides*, and *Neofusicoccum parvum* [18,19,20,21].

To elucidate the molecular mechanism underlying *B. fujianensis* infection in Chinese fir, we analyzed the transcriptomes of *B. fujianensis* cultured in PDA and inoculated onto Chinese fir shoots at different infection stages. This study provides a preliminary overview of candidate pathogenicity-related genes, laying the foundation for future functional validation and molecular dissection of the pathogen’s infection strategies.

## 2. Materials and Methods

### 2.1. RNA Extraction, Library Preparation, and Sequencing

Healthy shoots were collected from 2-year-old Chinese fir saplings (clone no. #061) growing in Yangkou State Forest Farm (26°49′18″ N, 117°53′30″ E, Nanping city Fujian, China). The collected shoots were first rinsed thoroughly with tap water to remove surface debris and subsequently surface-sterilized by immersion in 75% alcohol for 15 s, followed by three consecutive 30 s washes with sterile distilled water. The sterilized samples were the air-dried under a laminar-flow hood for 30–40 min before use. The *B. fujianensis* strain Cfsb3 was cultured on potato dextrose agar (PDA) at 25 °C for 4 days. PDA plugs (5 mm in diameter) containing actively growing mycelia were excised from the colony margin and inoculated onto the center of prepared Chinese fir shoots (one plug per shoot). After inoculation, shoots were placed in stainless steel trays lined with moist tissue paper, sealed with parafilm, and incubated at 25 °C. Samples were collected at 24 h (BGCF-E), 48 h (BGCF-M), and 5 d (BGCF-L) post inoculation, with three shoots collected per time point. Diseased shoot tips were excised, and transferred to sterilized 2 mL centrifuge tubes, immediately frozen in liquid nitrogen, and store it at −80 °C until RNA extraction. Mycelia of Cfsb3 grown on PDA were used as the control (BGPDA). All experiments were performed in triplicate to ensure reproducibility.

For RNA extraction, frozen samples were ground into fine powder under liquid nitrogen. Total RNA was extracted using the TransZol Up Plus RNA Kit (TransGen Biotech, Beijing, China) following the manufacturer’s instructions. RNA integrity was evaluated using the RNA Nano 6000 Assay Kit on a Bioanalyzer 2100 system (Agilent Technologies, Santa Clara, CA, USA). mRNA was purified from total RNA using oligo(dT)-conjugated magnetic beads. First-strand cDNA synthesis was synthesized using random hexamer primers and M-MuLV Reverse Transcriptase (RNase H^−^), followed by second-strand cDNA was synthesized using DNA Polymerase I and RNase H. DNA overhangs were blunted using the exonuclease and polymerase activity of DNA Polymerase I. After 3′-end adenylation, hairpin adapters were ligated to the cDNA fragments. Size-selected cDNA fragments (370~420 bp) was purified using the AMPure XP system (Beckman Coulter, Brea, CA, USA). Libraries were amplified with Phusion High-Fidelity DNA polymerase with universal and index primers. The amplified products were purified with AMPure XP beads, and library quality was evaluated using an Agilent 2100 Bioanalyzer (Agilent Technologies, Santa Clara, CA, USA). Indexed libraries were then clustered on a cBot Cluster Generation System (Illumina, San Diego, CA, USA) using the TruSeq PE Cluster Kit v3-cBot-HS (Illumina Inc., San Diego, CA, USA), following the manufacturer’s standardized protocol.

### 2.2. Data Quality Control and Analysis

Raw sequencing reads in FASTQ format were initially processed with custom Perl scripts for quality control. Low-quality reads were removed based on the following criteria: (i) reads containing adapter sequences, (ii) reads with poly-N stretches (>10% undetermined bases), or (iii) reads with more than 50% of bases with Phred quality scores below 20 (Q20). Sequence quality metrics, including Q20, Q30 scores, and GC content, were calculated for the filtered dataset using FastQC (v0.11.9). All downstream analyses were conducted exclusively on these high-quality clean reads. The reference genome index was constructed using HISAT2 (v2.0.5), and paired-end clean reads were aligned to the reference genome (*Bipolaris oryzae*: Index of /genomes/all/GCA/001/675/385/GCA_001675385.1_ASM167538v1, https://ftp.ncbi.nlm.nih.gov/genomes/all/GCA/001/675/385/GCA_001675385.1_ASM167538v1/, accessed on 21 October 2025).

### 2.3. GO and KEGG Enrichment Analysis

Differentially expressed genes (DEGs) were identified using the thresholds adjusted *p* (padj) < 0.05 and |log_2_foldchange| > 1. GO enrichment and KEGG enrichment analyses were conducted using clusterProfiler (v3.4.4). GO terms and KEGG pathways with significant DEG enrichment were identified based on the corrected *p*.

### 2.4. Validation of DEGs by RT-qPCR

RNA integrity and concentration were measured with a DeNovix DS-11 spectrophotometer (DeNovix Inc., Wilmington, DE, USA) after the total RNA samples were returned by the sequencing company (Novogene, Beijing, China) and treated with DNase I. The concentration of purified RNA was adjusted to 100 ng/µL. First-strand cDNA was synthesized using the HiScript^®^ III 1st Strand cDNA Synthesis Kit (+gDNA wiper) (Vazyme, Nanjing, China), using oligo(dT) primers in a 20-μL reaction system following the manufacturer’s instructions. To validate the RNA-seq results, RT-qPCR was performed using Taq Pro Universal SYBR qPCR Master Mix (Vazyme, Nanjing, China) on the QuantStudio^TM^ 6 Flex Real-time PCR system (Applied Biosystems, Waltham, MA, USA). Candidate pathogenicity-related genes were selected for validation, and actin served as the internal reference gene. Each reaction was performed with three biological replicates. Primers were designed using Primer Premier 6 software (Table 1). Relative gene expression levels were calculated using the 2^−ΔΔCt^ method, and data visualization was conducted accordingly.

## 3. Results

### 3.1. Transcriptome Sequencing Data Analysis

Transcriptome sequencing of BGPDA and BGCF samples generated more than 70 Gb of clean reads in total. The GC content of the reads ranged from 44.25% to 53.36%, with Q20 values > 97%, and Q30 values > 93%, indicating high sequencing accuracy. Clear reads were aligned to the *B. oryzae* genome (GCA_001675385.1_ASM167538v1) [22]. The map rate of the control group (BGPDA) to the genome was 87.41%, whereas BGCF samples (isolated from infected plant tissue) considerably much lower unique mapping rates (4.7–27.58%), likely due to extensive host RNA contamination and the use of a heterologous reference genome (Table 2). Although the uniquely mapped rates of BGCF samples were relatively low due to host RNA contamination and the use of a heterologous reference genome, differential expression analysis was still performed effectively by employing the BGPDA control (pure fungal culture) as the baseline. Strict filtering criteria (|log_2_FC| > 1, FDR < 0.05) were applied to genes with detectable fungal reads, ensuring that the identified DEGs reflect true pathogen-responsive signatures rather than background noise.

**Figure 1:**
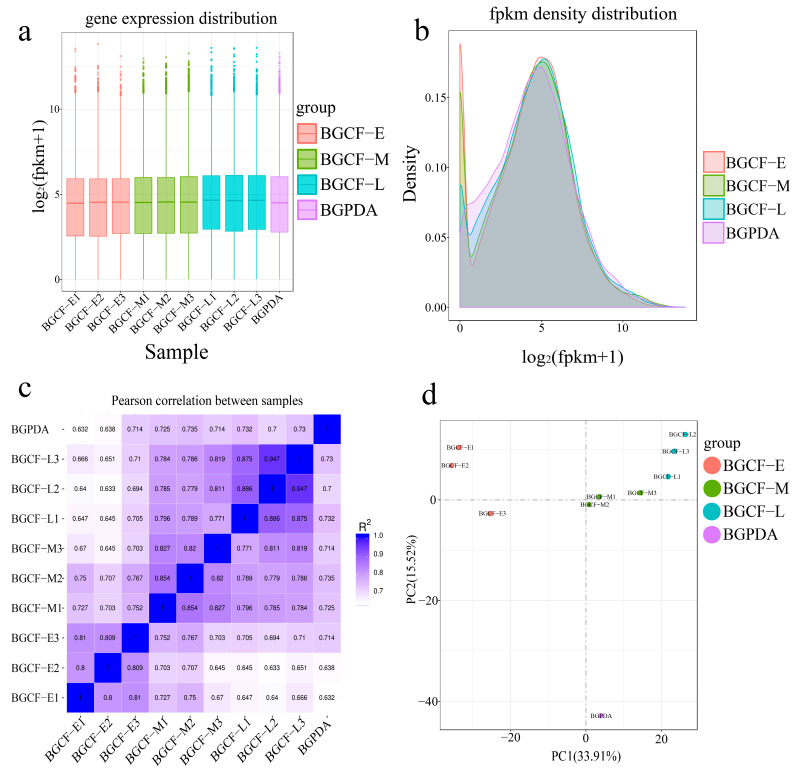
Analysis of gene expression of *B. fujianensis* at different infection times. (**a**) Box plot showing the distribution of gene expression levels across samples. (**b**) Density of gene expression levels in different samples. The *x*-axis represents log_2_(fpkm + 1), the *y*-axis represents density. (**c**) Heatmap of Pearson correlation coefficients among samples. (**d**) Principal component analysis (PCA) based on FPKM values of all samples, used to assess intergroup variation and intragroup reproducibility.

During sample analysis, a consistent trend in gene expression levels and gene density was observed. Based on Fragments Per Kilobase of transcript per Million mapped reads (FPKM) values, component analysis (PCA), and generated heatmaps, confirmed strong reproducibility. Pearson correlation coefficients (R^2^) were calculated to quantitatively evaluate the similarity of gene expression patterns within and between experimental groups. The square of the Pearson correlation coefficient (R^2^) values exceeded 0.8 for all samples pairs, indicating strong correlation between biological replicates. In the PCA plot, samples from different treatment groups showed significant dispersion at the level of principal component 1 (PC1) and principal component 2 (PC2), while replicates within the same group clustered tightly. These results confirmed both the rationality of the experimental design and the reliability of the transcriptomic data (Figure 1). Overall, the sequencing output met the quality requirements for downstream transcriptomic analysis.

### 3.2. Differentially Expressed Genes (DEGs)

**Figure 2:**
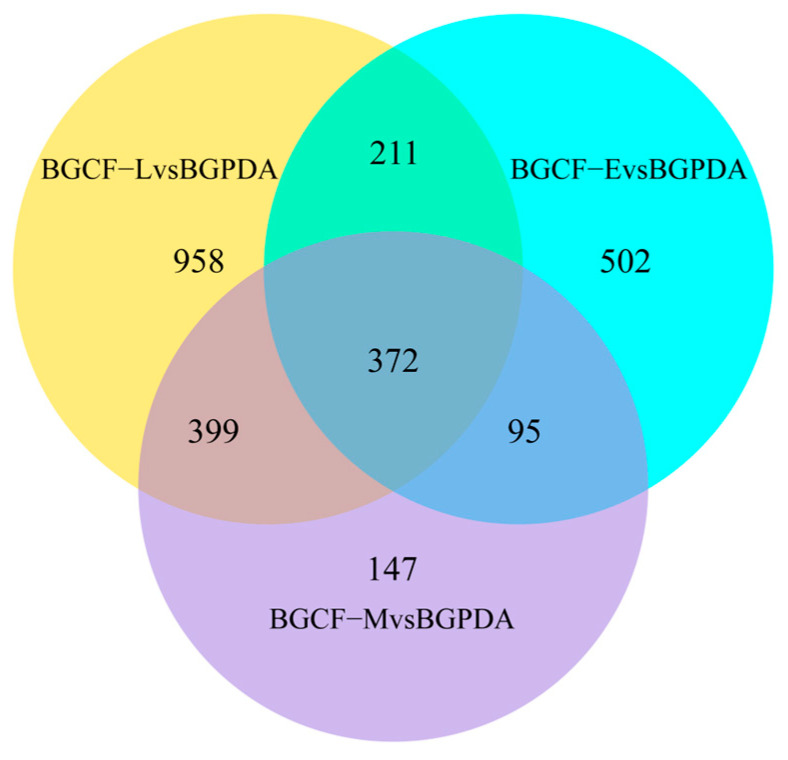
Venn diagram of transcripts expressed during cfsb3 growth on Chinese Fir vs. rich medium. Numbers of common and specific transcripts obtained in the transcriptome analysis of cfsb3 growing on Chinese Fir (BGCF) in comparison with its growth on Potato Dextrose Agar media (BGPDA). Unique transcripts are shown in only one of the two circles while shared transcripts are illustrated where the circles meet.

**Figure 3:**
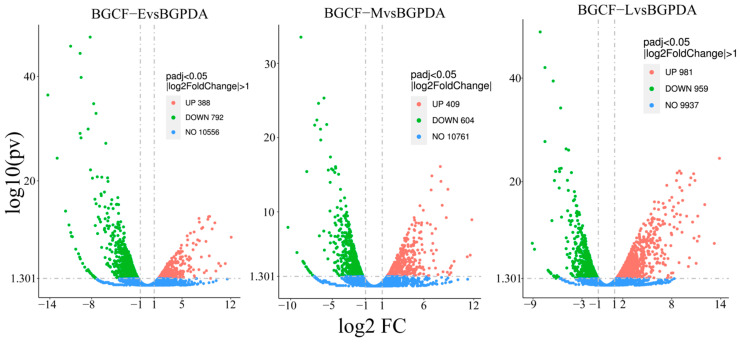
Volcano plots of differentially expressed genes (DEGs).

Pairwise comparisons between infected samples and the control identified significant transcriptional changes. A total of 2684 transcripts were detected including 392 shared across all conditions, while 502, 147, and 958 uniquely expressed at 24 h, 48 h, and 5 d, respectively (Figure 2). Compared with BGPDA, a total of 1180, 1013, and 1940 DEGs were detected at 24 h (BGCF-E), 48 h (BGCF-M), and 5 d (BGCF-L) post inoculation, respectively. Among them, 388 genes were upregulated and 792 downregulated at 24 h; 409 upregulated and 604 downregulated at 48 h; and 981 upregulated and 959 downregulated at 5 d. Notably, 372 DEGs were consistently upregulated across all three time points (Figure 3). These consistently upregulated genes are speculated to be associated with the pathogenicity and pathogenicity of *B. fujianensis* in Chinese fir.

### 3.3. Functional Annotation and Go Analysis

**Figure 4:**
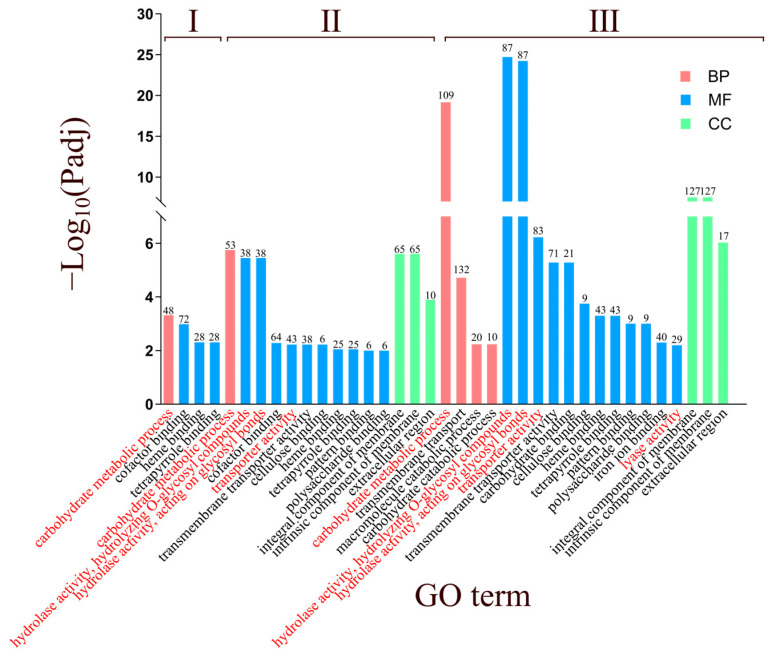
GO enrichment analysis of upregulated differentially expressed genes at three infection stage of *B. fujianensis*. Bars display significant enriched GO terms in the categories of biological process (BP), cellular component (CC), and molecular function (MF). The *x*-axis represents GO terms, and the *y*-axis shows the −log_10_(Padj) values. Different colors indicate BP (green), CC (blue), and MF (red). I, II, III, represent BGCF-E vs. BGPDA, BGCF-M vs. BGPDA, BGCF-L vs. BGPDA, respectively. Red Text Headings represent CFSB3 main infected enrichment items.

GO enrichment analysis revealed that DEGs from the three infection stages were mainly classified into molecular function (MF), biological process (BP), and cellular component (CC) categories (Figure 4). The majority of DEGs were enriched in MF terms, particularly hydrolase activity, hydrolyzing O-glycosyl compounds (GO:00,045,53) and hydrolase activity, acting on glycosyl bonds (GO:00,167,98), both of which showed progressively higher enrichment across infection stages. Notably, Lyase activity (GO:00,168,29) was specifically associated with the late stage, suggesting a role in advanced host tissue degradation.

In the BP category, enrichment was more evident at the late stage, especially in carbohydrate metabolic process (GO:00,059,75), transmembrane transport (GO:00,550,85), and macromolecule catabolic process (GO:00,090,57). Among these, the carbohydrate metabolic process (GO:00,059,75) showed the most pronounced increase in gene numbers compared with the early and middle stages. For the CC category, DEGs were mainly enriched in membrane-associated components (GO:00,160,21, GO:00,312,24) and the extracellular region (GO:00,055,76), indicating that *B. fujianensis* employs both membrane proteins and secreted effectors to interact with and invade host tissues.

Overall, these results suggest a dynamic infection strategy, where *B. fujianensis* progressively enhances carbohydrate-active enzyme activity, metabolic reprogramming, and secretion of virulence factors to colonize Chinese fir.

### 3.4. KEGG Enrichment Analysis of DEGs

**Figure 5:**
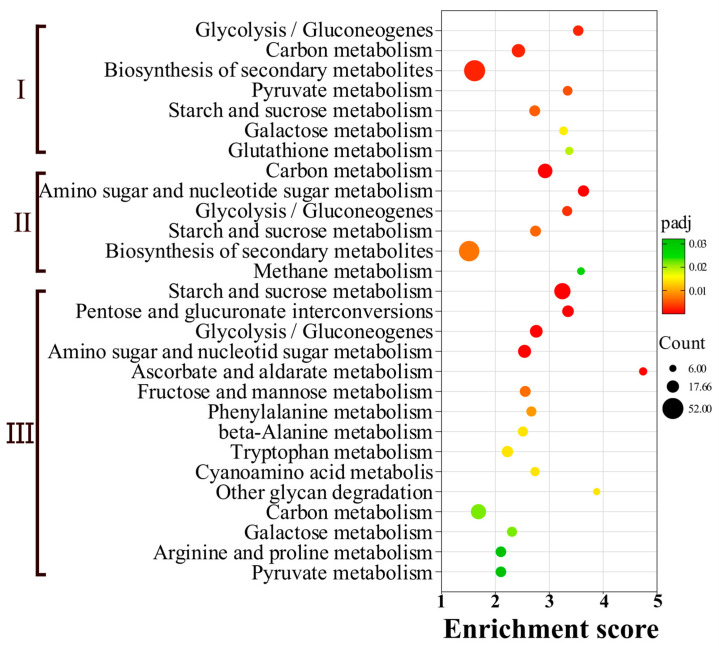
KEGG enrichment analysis of upregulated differentially expressed genes. Scatter plots are created to display the most significant KEGG pathways. In the graph, the horizontal axis represents the level of enrichment score of pathway enrichment, and the vertical axis represents the KEGG pathways. The size of the dots indicates the number of genes annotated to each KEGG pathway. The color gradient from red to green represents the level of significance of enrichment. I, II, III, stand for BGCF-E vs. BGPDA, BGCF-M vs. BGPDA, BGCF-L vs. BGPDA.

To investigate the functional roles of differentially expressed genes (DEGs) in metabolic pathways, KEGG pathway enrichment analysis was performed (Figure 4). Upregulated DEGs were significantly enriched in 28 metabolic pathways, involving a total of 478 genes. The major pathways included glycolysis/gluconeogenesis, biosynthesis of secondary metabolites, starch and sucrose metabolism, amino sugar and nucleotide sugar metabolism, pentose and glucuronate interconversions, and fructose and mannose metabolism (Figure 5). Notably, the overall biosynthesis of secondary metabolites contained the largest number of DEGs in early (BGCF-E vs. BGPDA, *n* = 52) and middle (BGCF-M vs. BGPDA, *n* = 49) stage, but was not significantly enriched in BGCF-L vs. BGPDA. This temporal shift suggests that *B. fujianensis* may rely on a broad spectrum of secondary metabolite production during the initial colonization and defense suppression, while later transitioning to other metabolic strategies to sustain host colonization.

Overall, the enrichment of carbohydrate metabolism, amino sugar pathways, and glycan degradation implies that pathogen invasion is closely associated with the secretion of toxins and cell wall–degrading enzymes, facilitating host tissue breakdown and successful infection.

### 3.5. Candidate Genes Associated with Pathogenesis

Among DEGs, 36 genes were identified as potentially involved in fungal pathogen–host interactions, including those encoding cell wall-degrading enzymes (CWDEs), proteases, fungal toxins, detoxification enzymes, and transport of toxic compounds. Of these, 20 genes were related to cell wall degradation (Table 3), with three genes (19126100, 19124354, 19126327) showing consistently upregulated expression with log2FC values greater than 5. Additionally, gene 19122268 exhibited exceptionally high expression (log_2_FC = 7.50) during early infection, suggesting its critical role in facilitating pathogen penetration. The expression levels of two key protease genes were significantly upregulated during fungal infection. Proteinase R exhibited sustained induction (log_2_FC = 3.56, 2.72, 2.16), while a putative aspergillopepsin A-like aspartic endopeptidase exhibited even higher expression (log_2_FC = 5.05, 3.19, 2.35), suggesting their roles in host tissue degradation and fungal pathogenicity (Table 4). Furthermore, genes involved in ophiobolin F biosynthesis were mainly upregulated during the late stage, indicating stage-dependent toxin production (Table 4). Quantitative analysis revealed that ophiobolin F exhibited no significant induction during initial and intermediate infection phases, but was strongly activated in late-stage, suggesting phase-dependent regulation of its virulence factor. Transcriptomic analysis also revealed significant induction of genes related to detoxification. Two homologs encoding sterigmatocystin biosynthetic P450 monooxygenase stcF (19126575 and 19117903) were strongly upregulated (13.85- and 10.34-fold, respectively), suggesting activation of this toxin biosynthetic pathway to enhance virulence or counteract host-derived oxidative stress. Other cytochrome P450 family members, such as paxP and ent-kaurene oxidase (3.12–3.83-fold), were also upregulated, supporting the central role of P450 enzymes in secondary metabolism and detoxification. Of particular interest, the upregulation of dehydrogenase patE (19117888: 6.89–7.42-fold) and cellobiose dehydrogenase (19122885: 3.40–4.90-fold) suggests their involvement in detoxification of host defensive metabolites via redox reactions (Table 4).

In addition, genes associated with the transport of toxin compounds were significantly induced. Notably, ABC transporter CDR4 and MFS-type transporter C1271.10c were highly upregulated, indicating that these transporters may facilitate toxin secretion or nutrient acquisition from the host. Concurrent induction of glucose/galactose transporters and inorganic phosphate transporter 1–2 further suggests metabolic reprogramming during host colonization. Moreover, the upregulation of putative glucose transporter rco-3 and ABC transporter G family member 11 highlights a coordinated regulation of multiple transport systems to counteract host-imposed stresses. Collectively, these findings provide new molecular insights into the pathogenic mechanisms of *B. fujianensis*, particularly the roles of CWDEs, toxins, detoxification enzymes, and transporters in successful host infection (Table 4).

### 3.6. Expression Validation of Pathogenicity-Related Genes

**Figure 6:**
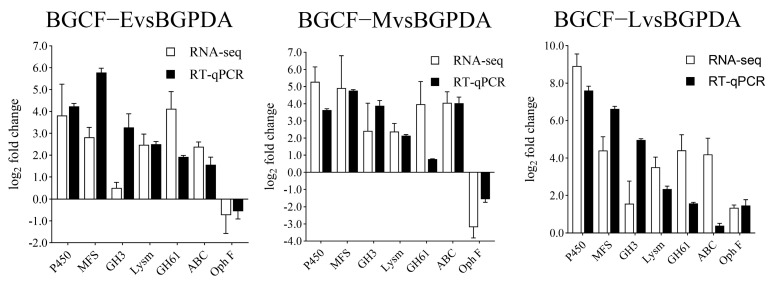
RT-qPCR validation of selected pathogenicity-related genes in *B. fujianensis*.

To validate the RNA-seq data and assess the temporal expression patterns of pathogenicity-related genes, RT-qPCR analysis was performed on seven selected DEGs involved in the infection pathway. The results showed that the expression patterns of these genes were highly consistent with the RNA-Seq data, confirming the reliability of transcriptomic analysis (Figure 6).

Genes encoding cell wall-degrading enzymes (e.g., glycoside hydrolase family proteins, GH3, GH61) and proteases (e.g., Proteinase R, aspergillopepsin A-like aspartic endopeptidase) were significantly upregulated at the early and middle infection stages, supporting their roles in host tissue penetration and degradation. By contrast, genes associated with ophiobolin F (oph F) biosynthesis exhibited marked induction during the middle and late infection stage, indicating stage-specific toxin production. Detoxification-related genes (e.g., P450 monooxygenases, dehydrogenases) and transporters (e.g., ABC and MFS transporters) displayed progressive upregulation, suggesting their involvement in counteracting host-derived stress and facilitating nutrient acquisition. Together, these results corroborate the transcriptomic analysis and highlight the temporal regulation of multiple pathogenicity-related processes in *B. fujianensis*.

## 4. Discussion

Understanding the molecular pathogenesis of *B. fujianensis* is crucial for developing effective control strategies against Chinese fir shoot blight (CFSB). In this study, we performed comprehensive transcriptomic analysis of *B. fujianensis* at different infection stages and validated selected DEGs by RT-qPCR. The consistent expression patterns between RNA-seq and RT-qPCR confirmed the reliability of the transcriptome data, providing a solid foundation for dissecting the infection mechanism.

*B. fujianensis* is phylogenetically closely to *B. oryzae* and may represent an evolutionary derivative of the latter that has adapted to Chinese fir [16]. Therefore, the *B. oryzae* genome (ASM167538v1, https://ftp.ncbi.nlm.nih.gov/genomes/all/GCA/001/675/385/GCA_001675385.1_ASM167538v1/, accessed on 21 October 2025) was selected as the reference for downstream analyses. Our transcriptomic analysis revealed substantial transcriptional reprogramming during host infection. The differential gene expression analysis revealed extensive variation among the three experimental groups, indicating significant differences in gene expression patterns across distinct experimental conditions. As the infection progressed over time, the number of GO enriched terms showed an increasing trend. Among these, certain GO terms merit particular attention, including “carbohydrate metabolic process,” “transporter activity,” “hydrolase activity, acting on glycosyl bonds,” “lyase activity,” and “cofactor binding.” These findings underscore key biological processes and molecular functions potentially implicated in the infection process.

KEGG enrichment analysis further highlighted secondary metabolite biosynthesis, carbon metabolism, and glycolysis/gluconeogenesis as major pathways. Carbohydrates serve as essential energy sources for pathogen growth and infection.

A considerable body of research has indicated that fungal cell wall-degrading enzymes (CWDEs) are pivotal virulence factors for plant pathogens [23,24,25]. The upregulation of carbohydrate metabolism of glycoside hydrolases (GHs), polysaccharide lyases (PLs), and LysM proteins suggests that *B. fujianensis* employs cell wall-degrading enzymes (CWDEs) as key virulence factors during infection. These enzymes under scrutiny in this study are capable of competitively binding free chitin oligosaccharides and thereby impeding their interaction with host receptors. This inhibition of chitin-induced host immune responses is a key mechanism through which the enzymes exert their biological function. This mechanism is vital for protecting hyphae from chitinase-mediated hydrolysis, thus ensuring its crucial role in the process of pathogenicity [26,27].

A particular important observation was the upregulation of ophiobolin biosynthetic genes. Ophiobolins are well-characterized phytotoxins with multiple modes of action: (i) inhibition of calcium–calmodulin signaling in host cells, leading to cellular dysfunction and necrosis; (ii) inhibition of β-1,3 glucan synthase by ophiobolin A, disrupting cell wall biosynthesis; and (iii), alteration of plasma membrane permeability, resulting in electrolyte leakage, impaired photosynthesis, and inhibition of protein and nucleic acid synthesis [28,29]. Intriguingly, despite a decline in pathway-level signaling, the ophiobolin F biosynthetic gene cluster itself exhibited a significant transcriptional surge during late infection. This stage-specific activation suggests that the fungus prioritizes toxin synthesis over general secondary metabolism after host establishment, thereby facilitating aggressive tissue necrosis and disease progression. Such a “broad-to-specific” temporal switch highlights a finely tuned regulatory program in which *B. fujianensis* escalates virulence by precisely timing toxin release rather than maintaining an energetically costly global secondary metabolite arsenal. We also observed strong upregulation of cytochrome P450 monooxygenases and MFS transporters, both of which are closely linked to fungal pathogenicity. P450 enzymes participate in secondary metabolite biosynthesis, detoxification of host-derived compounds, and oxidative stress tolerance [30,31,32]. These findings provide critical insights into the molecular mechanisms underlying CFSB3 infection.

P450 enzymes are evolutionarily conserved across the fungal kingdom and have consistently been implicated in colonization efficiency and virulence-factor expression in phytopathogens [33]. In cfsb3, P450 genes constitute an expansive family that clusters phylogenetically with orthologues from plant-infecting fungi [34,35]. In *Sporisorium scitamineum*, the CYP isoform *SsCyp86* integrates fatty-acid metabolism with oxidative-stress responses, sexual development, and pathogenicity by governing the transcription factor SsPRF1 [36]. The coordinated induction of multiple P450 genes in *B. fujianensis* suggests a similarly multifaceted role during infection. Concurrent up-regulation of MFS transporters—known to mediate toxin efflux, drug resistance, and adaptation to host defense metabolites [30,31,32]—further underscores their collective contribution to virulence and host adaptation.

Our results suggest that *B. fujianensis* may exhibit stronger virulence on *C. lanceolata* than *B. oryzae*, potentially due to differences in CWDEs activity or potential variations in toxin structure. Future studies, including genome sequencing and bioinformatics predictions of CWDEs and toxin, together with experimental validation, could provide further insights into the pathogenic mechanisms of *B. fujianensis*.

## 5. Conclusions

Our findings indicate that *B. fujianensis* employs a multi-tiered infection strategy involving: (i) early activation of CWDEs to breach host cell walls, (ii) secretion of phytotoxins (e.g., ophiobolins) to impair host physiology, and (iii) upregulation of detoxification enzymes and transporters to counter host defense compounds. This integrated pathogenic program reflects the adaptive evolution of *B. fujianensis* to its woody host. By combining transcriptomic analysis with functional annotation, we provide a preliminary molecular framework for understanding how this novel pathogen colonizes Chinese fir. These candidate pathogenicity-related genes offer promising targets for future functional validation, resistance breeding, and disease management strategies.

## Figures and Tables

**Table 1:** Primers used for RT-qPCR analysis of candidate pathogenicity-related genes.

Genes ID	Description	Primer Sequence 5′-3′ (F; R)
19117903	Cytochrome P450	CCATCCTCCAATTCTCGCTCCT
		GCAACTTCCTCGCCCTGTAGA
19117919	MFS-type transporter	TGGCTCCGTCATCCTCCTCT
		GTTGTGGGCGTTGTGTGGTAG
19116538	Glycoside hydrolase family 3 protein	GGAGAAGATGCTGGCGAGAACC
		CGCTTGATGGCTTCGAGAGGAG
19119884	LysM domain-containing protein	GTTGCCGACTCCATCACCATTG
		GCCGCACAAGACGACGAATAC
19124682	Glycoside hydrolase-61	CGGCACTGGCGACATCATCT
		TAGGAAGCAGCGTAGGTGAGGA
19119147	ABC transporter	TCTGCGGCGGAATCATCACTT
		TGCGAAGAAGACACCGTTGAGT
19119514	Ophiobolin F synthase	ACTACTGGTGCTCGGCTTGC
		GCGAAGGCTCATCATCACTGGA
Reference gene	Actin	TATGGGCCAAAAGGACTCA
		CACGCAGCTCGTTGTAGAAG

**Table 2:** Summary of Illumina sequence reads obtained from BGPDA and BGCF.

	Sample	Clean Reads	Clean Bases	Unique Map	Q20 (%)	Q30 (%)	GC pct (%)
BGPDA	BGPDA	43773234	6.57 G	38,260,232 (87.41%)	97.67	93.65	53.36
BGCF-E	E1	41553844	6.23 G	19,533,51 (4.7%)	97.84	93.54	44.64
	E2	42628686	6.39 G	99,231,4 (2.33%)	97.84	93.52	44.25
	E3	40086040	6.01 G	26,738,55 (6.67%)	97.76	93.36	44.81
BGCF-M	M1	44515990	6.68 G	36,054,76 (7.97%)	97.76	93.36	45.01
	M2	42124290	6.32 G	11,495,698 (27.58%)	97.81	93.49	45.22
	M3	43925140	6.59 G	77,371,51 (18.71%)	98.03	94.08	44.86
BGCF-L	L1	45264002	6.79 G	24,171,85 (5.43%)	97.79	93.47	45.65
	L2	41676764	6.25 G	21,130,60 (5.02%)	97.78	93.56	48.55
	L3	41358898	6.2 G	26,293,76 (5.99%)	97.89	93.78	47.57

Note: BGPDA; *B. fujianensis* grown on PDA; BGCF; E1–E3: *B. fujianensis* grown on Chinese fir shoot for 24 h, 48 h (M1–M3), 5 days (L1–L3); Q20: The percentage of bases with phred value greater than 20 in total bases; Q30: The percentage of bases with phred value greater than 30 in total bases; GC pct: The percentages of G and C in the four bases of clean reads.

**Table 3:** *B. fujianensis* genes encoding cell wall degrading enzymes.

Gene ID	Description	E-log_2_FC	M-log_2_FC	L-log_2_FC
Cell Wall Degrading Enzymes				
19122268	glycoside hydrolase family 18 protein	7.50	6.99	5.92
19126100	glycoside hydrolase family 62 protein	4.82	5.53	8.60
19124354	glycoside hydrolase family 10 protein	3.08	5.39	6.24
19126327	glycoside hydrolase family 18 protein	4.78	5.15	5.32
19118171	glycoside hydrolase family 131 protein	2.77	4.84	3.25
19119168	glycoside hydrolase family 10 protein	3.14	4.56	3.13
19125825	glycoside hydrolase family 71 protein	4.44	4.48	1.88
19121668	glycoside hydrolase family 18 protein	5.99	4.17	5.07
19126538	glycoside hydrolase family 3 protein	2.39	4.12	4.35
19120137	glycoside hydrolase family 15 protein	2.51	1.88	2.95
19126692	glycoside hydrolase family 31 protein	2.26	1.85	1.83
19125121	glycoside hydrolase family 20 protein	2.25	1.80	2.24
19122960	Probable endo-beta-1,4-glucanase D	3.96	4.45	5.16
19124682	Cellulase-61a	3.11	5.94	5.11
19125385	Probable endo-beta-1,4-glucanase D	2.98	2.28	1.64
19122221	Probable endo-beta-1,4-glucanase D	2.84	4.49	8.98
19129443	Probable endo-beta-1,4-glucanase D	2.81	6.88	5.47
19127491	Probable endo-beta-1,4-glucanase D	2.39	3.60	1.49
19122124	Cutinase transcription factor 1 beta	2.73	3.19	3.60
19127443	polysaccharide lyase family 1 protein	2.15	2.92	1.63

**Table 4:** Genes of *B. fujianensis* potentially involved in pathogenesis.

Gene ID	Description	E-log_2_FC	M-log_2_FC	L-log_2_FC
Genes Related to Proteases				
19124929	Proteinase R	3.56	2.72	2.16
19125779	Putative aspergillopepsin A-like aspartic endopeptidase	5.05	3.19	2.35
Genes Related to Toxins Production				
19119514	Ophiobolin F	−1.51	−2.93	1.90
Genes Related to Detoxification of Toxic Compounds				
19126575	Probable sterigmatocystin biosynthesis P450 monooxygenase stcF	6.84	7.12	13.85
19117903	Probable sterigmatocystin biosynthesis P450 monooxygenase stcF	4.18	6.78	10.34
19128488	Cytochrome P450 monooxygenase paxP	3.12	3.83	3.67
19128498	Ent-kaurene oxidase	2.59	2.79	2.79
19117888	Dehydrogenase patE	7.42	6.65	6.89
19122885	Cellobiose dehydrogenase	3.69	4.90	3.40
19124701	Dehydrogenase patE	3.61	2.71	1.90
Genes Related to Transport of Toxic Compounds				
19119582	Glucose/galactose transporter	4.10	4.32	2.61
19124715	ABC transporter CDR4	3.47	4.20	6.60
19128823	Probable glucose transporter rco-3	3.46	3.71	2.02
19117919	MFS-type transporter C1271.10c	2.53	2.40	3.54
19125740	Inorganic phosphate transporter 1–2	2.51	3.52	2.40
19118321	ABC transporter G family member 11	2.32	2.23	2.86

## Data Availability

The raw transcriptome sequencing data generated in this study have been deposited in the NCBI Sequence Read Archive (SRA) under BioProject ID PRJNA1321556. The data include 10 BioSamples (SAMN51234593–SAMN51234602) corresponding to different experimental conditions. All data are publicly accessible at: https://www.ncbi.nlm.nih.gov/bioproject/PRJNA1321556 (accessed on 21 October 2025).

## Abbreviations

The following abbreviations are used in this manuscript:

RNA	Ribonucleic Acid
DNA	Deoxyribonucleic Acid
DEGs	Differentially expressed genes
GO	Gene ontology
KEGG	Kyoto encyclopedia of genes and genomes
GHs	Glycoside hydrolases
PCA	Principal component analysis
FPKM	Fragments per kilobase of exon model per million mapped fragments
PDA	Potato dextrose agar
RT-qPCR	Reverse Transcription quantitative Polymerase Chain Reaction
NCBI	National center for biotechnology information
